# Lung Cancer Management with Silibinin: A Historical and Translational Perspective

**DOI:** 10.3390/ph14060559

**Published:** 2021-06-11

**Authors:** Sara Verdura, Elisabet Cuyàs, Verónica Ruiz-Torres, Vicente Micol, Jorge Joven, Joaquim Bosch-Barrera, Javier A. Menendez

**Affiliations:** 1 Girona Biomedical Research Institute (IDIBGI), 17190 Girona, Spain; sverdura@idibgi.org (S.V.); ecuyas@idibgi.org (E.C.); 2 Metabolism and Cancer Group, Program against Cancer Therapeutic Resistance (ProCURE), Catalan Institute of Oncology, 17007 Girona, Spain; 3 Instituto de Investigación, Desarrollo e Innovación en Biotecnología Sanitaria de Elche (IDiBE) and Instituto de Biología Molecular y Celular (IBMC), Universidad Miguel Hernández (UMH), 03202 Elche, Spain; vruiz@umh.es (V.R.-T.); vmicol@umh.es (V.M.); 4 Unitat de Recerca Biomèdica (URB-CRB), Hospital Universitari de Sant Joan, Institut d’Investigació Sanitària Pere Virgili, Universitat Rovira i Virgili, 43201 Reus, Spain; jjoven@grupsagessa.com; 5 Medical Oncology, Catalan Institute of Oncology, Dr. Josep Trueta Hospital of Girona, 17007 Girona, Spain; 6 Department of Medical Sciences, Faculty of Medicine, University of Girona (UdG), 17003 Girona, Spain

**Keywords:** silibinin, silymarin, non-small cell lung cancer, EMT, metastasis, STAT3

## Abstract

The flavonolignan silibinin, the major bioactive component of the silymarin extract of *Silybum marianum* (milk thistle) seeds, is gaining traction as a novel anti-cancer therapeutic. Here, we review the historical developments that have laid the groundwork for the evaluation of silibinin as a chemopreventive and therapeutic agent in human lung cancer, including translational insights into its mechanism of action to control the aggressive behavior of lung carcinoma subtypes prone to metastasis. First, we summarize the evidence from chemically induced primary lung tumors supporting a role for silibinin in lung cancer prevention. Second, we reassess the preclinical and clinical evidence on the effectiveness of silibinin against drug resistance and brain metastasis traits of lung carcinomas. Third, we revisit the transcription factor STAT3 as a central tumor-cell intrinsic and microenvironmental target of silibinin in primary lung tumors and brain metastasis. Finally, by unraveling the selective vulnerability of silibinin-treated tumor cells to drugs using CRISPR-based chemosensitivity screenings (e.g., the hexosamine biosynthesis pathway inhibitor azaserine), we illustrate how the therapeutic use of silibinin against targetable weaknesses might be capitalized in specific lung cancer subtypes (e.g., *KRAS/STK11* co-mutant tumors). Forthcoming studies should take up the challenge of developing silibinin and/or next-generation silibinin derivatives as novel lung cancer-preventive and therapeutic biomolecules.

## 1. Introduction

Phytochemicals are biologically active compounds synthesized by plants (*Phyto* means “plant” in Greek). The term, however, is generally employed for those influencing human health. Flavonoids are a subclass of polyphenol phytochemicals that are commonly present in fruits, vegetables, nuts, seeds, herbs, spices, stems, flowers, teas, and red wine [1,2]. As they have existed in nature for millions of years, flavonoids have a long historical association with animal species throughout evolution, which likely explains their myriad biochemical and pharmacological properties [3]. Although not without limitations, the mutualistic relationship between plant flavonoids and animals, which is embraced in the concept of xenohormesis [4,5], can be applied to human pathophysiology; in particular, the various bioactivities of flavonoids (e.g., anti-inflammatory, antioxidant, antiallergic, hepatoprotective, antithrombotic, antiviral, and anticarcinogenic) in numerous biological systems. 

Flavonolignans are a minor subclass of flavonoids comprising a flavonoid moiety and a lignan (phenylpropanoid) part. They were first isolated from the seeds of milk thistle (*Silybum marianum* (L.) Gaertn.), an annual/biannual plant of the *Asteraceae* family flowering in July–August with characteristic reddish-purple flowers. The milk thistle is indigenous to South Europe, South Russia, Asia Minor, and North Africa, but has also been naturalized in North and South America and in South Australia. The so-called silymarin extract of milk thistle, which was classified by the World Health Organization as an official medicine with health-promoting properties in the 1970s, is obtained through organic solvent extraction and represents 1.5–3% of the dry weight of the fruit. Silymarin contains a mixture of flavonolignans of mainly four isomers: silibinin (or silybin), isosilybin, silychristin, and silydianin. There is also a minor fraction of polymeric and oxidized polyphenolic components [6,7,8,9,10,11,12], including two pairs of diastereomers––silibinin A/B and isosilybin A/B. Silibinin is composed of a 1:1 mixture of silibinin A and B and comprises 50–70% of the extract and 20–40% of the commonly used pharmaceutical preparations [11,13,14]. Whereas the chemical composition of milk thistle fruits includes other flavonoids (e.g., taxifolin, quercetin, kaempferol, apigenin), the highest concentration of silymarin corresponds to silibinin, which is considered the major bioactive component [15,16,17,18].

**Figure 1:**
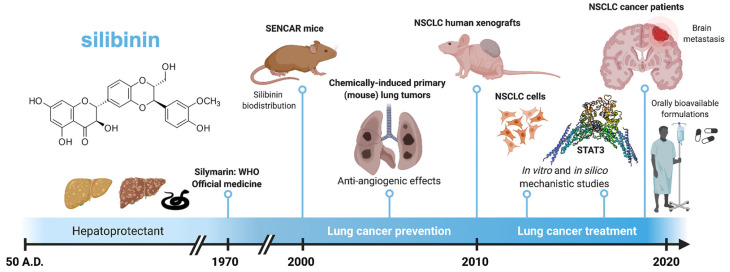
Key milestones in the timeline of silibinin research in lung cancer. Originally employed as a hepatoprotectant and a remedy for the bites of poisonous snakes hundreds of years ago, silibinin has recently demonstrated significant clinical activity in patients with non-small cell lung cancer and brain metastases when used in new orally bioavailable formulations. Created with BioRender.

Originally described as a cure for the venom of poisonous snakes, silibinin is the most extensively studied flavonolignan and is currently clinically employed to treat amatoxin/*Amanita* mushroom poisoning or lipotoxic injury in fatty liver diseases. Here, we review the historical context of the development of silibinin research in lung cancer (Figure 1). A literature search (silibinin AND lung cancer) was initially conducted in the electronic database PubMed with no date-range restriction. No quality-assessment scale systems were used to evaluate the collected studies. Manuscripts were screened by checking the title and abstract or reading the full text to determine their inclusion. In addition, we provide some experimental results to illustrate how we might capitalize on the therapeutic use of silibinin against targetable weaknesses in specific subgroups of patients with lung cancer.

## 2. Silibinin-Containing Milk Thistle Fruits and Human Health: A Brief Historical Overview

The name milk thistle originates from a legend that Mary, when leaving for Egypt with the infant Jesus, found shelter in a bower formed from the thorny leaves of *S. marianum*. While nursing Jesus, she spilled some breast milk onto the plant, and this resulted in the characteristic milky-white veins of the plant’s leaves.

Milk thistle fruits have been used for over 2000 years in the treatment of liver- and biliary-related diseases. While the first record of *S. marianum* can be found in the Old Testament (Genesis 3:18), it had already been used in ancient Greece and in millenarian Indian and Chinese medicines to resolve liver and gallbladder problems. Theophrastus of Eresos (fourth century B.C.), Pedanios Dioscorides (50 A.D.), and Plinius the Elder (first century A.D.) were the first to report the medicinal benefits of milk thistle fruits. In his work “*De Materia Medica*”, Dioscorides described *S. marianum* as a remedy for the bites of poisonous snakes and for melancholic depression, which was believed to be a “liver complaint” at that time.

Used in the Middle Ages as an antidote for liver toxins, renaissance and humanistic naturalists and physicians included milk thistle in their herbal medicine armamentarium. Native American Indians, 19th century physicians, and herbalists also employed preparations of milk thistle fruits to treat a variety of diseases, particularly liver pathologies. In the last 40–50 years, the use of silibinin-dependent, bioactive silymarin extracts for treatment of liver disorders such as alcoholic liver disease, nonalcoholic liver disease, drug-induced liver injury, cirrhosis, viral hepatitis, and mushroom poisoning has been well documented [12,18,19]. Patients with liver disorders treated with silymarin show a more rapid improvement in liver function than those receiving placebo. Likewise, in patients with alcoholic liver cirrhosis, administration of silymarin for several years resulted in a significantly reduced mortality rate [15,20]. Not surprisingly, silymarin is one of the most frequently sold dietary supplements for hepatitis and cirrhosis in the USA and Europe [21].

## 3. Silibinin to Therapeutically Manage Lung Cancer: Pioneering Studies

Dr. Agarwal and colleagues at the University of Colorado Health Sciences Center (Denver, USA) pioneered the investigation of silibinin to prevent and treat human malignancies in different experimental models of skin [22,23], prostate [24,25], and lung [26,27] cancer. Based on the strong antioxidant activity of silymarin and the fact that it was already in clinical use for a range of liver, gall bladder, and even dermatological conditions [28], they conducted a series of cancer-centered studies with silymarin in both short-term cell culture and long-term animal models. Using SENCAR mice, which are highly sensitive to tumor initiation and promotion in response to carcinogens and promoters [29,30], they initially assessed the tissue biodistribution and conjugate formation of systematically administered silibinin in different mouse tissues and its effect on phase II detoxification enzymes [26]. They found that silibinin could rapidly distribute as both free and conjugated forms and significantly induced phase II enzymes in the tissues examined. These findings strongly suggested that silibinin might reach target organs to exert anti-cancer effects, providing the first basis to evaluate the cancer preventive and interventive effects of silibinin in experimental models of carcinogenesis [26]. Using established cell models of small cell (SCLC) and non-small cell (NSCLC) lung carcinoma, the Agarwal group was the first to report that micromolar concentrations of silibinin could significantly increase growth inhibition, cell cycle arrest, and apoptotic cell death [31], warranting further studies to establish the efficacy and mechanism(s) of action of silibinin as a non-toxic therapeutic agent in additional lung tumor models.

## 4. Silibinin and Lung Cancer Prevention: Evidence from Chemically Induced Primary Lung Tumors

The Agarwal group demonstrated that oral silibinin (200 mg/kg, 5 d/wk for 33 days) inhibited NSCLC A549 xenograft tumor growth and suppressed the systemic toxicity of co-administered doxorubicin in athymic BALB/c *nu/nu* mice through a mechanism likely dependent on the regulation of nuclear factor kappaB (NFκB), a key player in the chemoresistance and dose-related (acute and cumulative) toxicity of anthracyclines [32]. In contrast to these findings, Yan and colleagues reported the failure of 0.05% and 0.1% silibinin in the diet (*wt*/*wt*) to significantly reduce tumor multiplicity and load in a mouse model of tobacco-driven lung carcinogenesis [33]. In another study by the Agarwal group, the lack of efficacy of silibinin in preventing benzo(a)pyrene-induced pulmonary adenoma formation and growth reported in the aforementioned Yan study was not observed when the effects of dietary silibinin (0–1% *wt*/*wt*) on the growth, progression, and angiogenesis of lung tumors induced by urethane (a carcinogenic contaminant of alcoholic beverages and other fermentation products) were tested in A/J mice [34]. Chronic oral consumption of silibinin significantly lowered lung tumor multiplicity, prevented lung tumors from growing beyond a small size (in a dose-dependent fashion), and blunted tumor angiogenesis, a plausible mechanism contributing to the efficacy of silibinin in this model [34].

Mechanistically, the cancer-preventive activity of silibinin was initially attributed to the reduced lung tumor expression of the angiogenic factor vascular endothelial growth factor (VEGF), mediated by the suppression of VEGF regulators such as cyclooxygenase-2 (COX2) and inducible nitric oxide synthase (iNOS) [34]. Silibinin appeared to target multiple cytokine (IFNγ, IL-1β, and TNF-α)-induced signaling pathways such as the signal transducer and activator of transcription 3 (STAT3) to ultimately lower COX2 and iNOS expression in lung cancer cells [35,36]. When the chemotherapeutic effects of oral silibinin on the growth and progression of established, urethane-induced, lung adenocarcinomas in A/J mice were studied, its strong ability to suppress both tumor number and size correlated with a reduced antiangiogenic activity mediated by decreased cytokine production in tumor-associated macrophages and suppression of NFκB and STAT3 activation in lung cancer cells [36]. Importantly, the capacity of silibinin to prevent urethane-induced lung tumorigenesis in mice was completely lost upon genetic ablation of *Nos2* (iNOS) [37], strongly suggesting that silibinin exerts its chemopreventive and angiopreventive effects through blockade of iNOS expression in lung tumors. Careful examination of the mechanism of action of silibinin on cell signaling elicited by a cytokine mixture (IFNγ + TNF-α) in tumor-derived LM2 mouse lung epithelial cells revealed that its ability to regulate the expression of metalloproteinases and the angiogenesis drivers COX2 and iNOS was causally mediated through impairment of STAT3 activation and nuclear localization [38]. As no 50% lethal dose (LD_50_) has been reported in laboratory animals, and silibinin treatment has been considered exceptionally safe after acute or long-term chronic administration in both animals and humans, these findings strongly supported the investigation of silibinin as a chemopreventive agent for suppressing lung tumor growth and progression in humans [27].

## 5. Silibinin and Lung Cancer Treatment: Evidence from Laboratory In Vitro and Animal Models

An ever-growing number of studies have tested the capacity of silibinin to exert inhibitory activities against cultured cancer cells and tumor xenografts, to enhance the efficacy of other therapeutic agents (reviewed in [39,40]), and to block the emergence of cancer drug resistance in pre-clinical models of lung cancer, including those involving NSCLC-targeted therapies such as epidermal growth factor receptor (EGFR)- and anaplastic lymphoma kinase (ALK)-tyrosine kinase inhibitors (TKIs).

### 5.1. Silibinin and Lung Cancer Drug Resistance

Early studies evaluating silibinin against established cell lines representative of different NSCLC subtypes revealed that micromolar concentrations significantly inhibited cell proliferation by inducing cell cycle arrest and modulating multiple cell cycle regulators, including cyclin-dependent kinases and their corresponding cyclins [41,42]. In later studies, we and others described the capacity of silibinin to exert cytostatic, cytotoxic, and apoptotic effects in various NSCLC cell models [43,44,45]. Importantly, silibinin could restore drug sensitivity to NSCLC cells with acquired resistance to EGFR- and ALK-TKIs in vitro and in vivo.

Rho and colleagues investigated whether the addition of silibinin to EGFR-targeted therapy using first-generation EGFR-TKIs (gefitinib or erlotinib) could overcome primary and acquired resistance due to the presence of the *EGFR T790M* mutation [46]. They found that silibinin enhanced the ability of EGFR-TKIs to downregulate EGFR signals by inhibiting receptor dimerization of EGFR family members (EGFR, HER2, and HER3) in vitro. Moreover, the combination silibinin and erlotinib suppressed tumor growth in erlotinib-resistant (*EGFR T790M*) PC-9 NSCLC xenografts [46]. The ability of silibinin to resensitize NSCLC cells to EGFR- and ALK-TKIs occurs even in the absence of secondary *EGFR* mutations. Using gefitinib- and erlotinib-refractory NSCLC cell models in which EGFR-TKI resistance occurs via the activation of bypass survival signals with other receptor tyrosine kinases (e.g., hyperactive insulin-like growth factor-1 receptor [IGF-1R]) [47] and/or epithelial-to-mesenchymal transition (EMT) [48,49], a water-soluble form of silibinin complexed with the amino-sugar meglumine could efficiently restore EGFR-TKI sensitivity in NSCLC mouse xenografts [48,49]. Mechanistically, silibinin could differentially eliminate cancer stem cell (CSC)-like cells within EGFR-TKI-refractory heterogeneous NSCLC populations with aldehyde dehydrogenase isoform 1 (ALDHA1) overexpression and self-renewal capacity [43,50]. Using a model of ALK-translocated NSCLC in which acquired refractoriness to the ALK-TKI crizotinib was driven by activation of TGFβ-induced EMT in the absence of secondary mutations in the kinase domain of ALK, silibinin-induced inhibition of STAT3 was found to synergistically interact with crizotinib to reverse acquired resistance and restore sensitivity in crizotinib-resistant cells [46].

**Figure 2:**
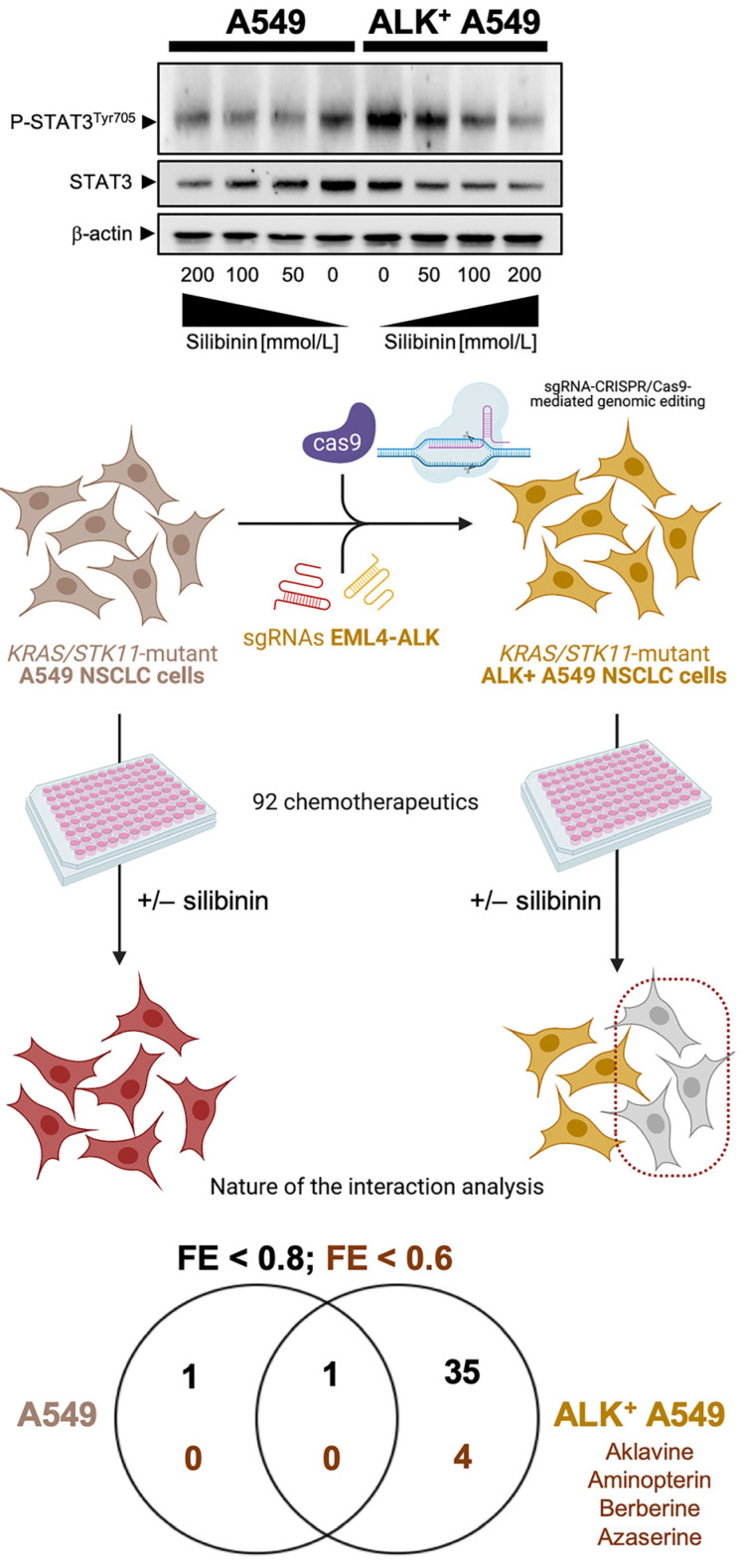
EML4-ALK-dependent chemosensitizing effects of silibinin in non-small cell lung cancer cells. We utilized the Phenotypic Microarray system, marketed and sold by Biolog (www.biolog.com, access date: 30 May 2021) to measure the sensitivity of an A549 non-small cell lung cancer (NSCLC) cell line with an *EML4-ALK* fusion isogenic oncogene (https://www.nature.com/articles/d42473-019-00011-z, access date: 30 May 2021) to a variety of growth inhibitors (in total, 92) in microplates (PM-M11 to PM-M14). This approach enables the simultaneous testing of tens of phenotypes and the identification of shared versus selective sensitivities to a wide variety of mechanistically distinct drugs. We chose a silibinin concentration of 100 µmol/L, which was notably lower than the IC_50_ value against A549 cells and consistently reduced cell viability by less than 5% in multiple experiments using the colorimetric redox-sensitive dye employed in the Biolog technology. A set of “negative” control plates cultured in the presence of the silibinin vehicle DMSO were used to assess the inherent response of A549/ALK^+^ A549 cells to growth inhibitors. A set of “positive” plates cultured in the presence of 100 µmol/L silibinin served to assess the nature of the interaction between silibinin and the 92 drugs pre-loaded in the 96-well plates (4 graded concentrations/each). We assessed the nature of the cytotoxic responses based on synergistic, additive, or antagonistic categories using an arbitrarily defined ratio of observed effect/theoretical effect, the so-called fractional effect (FE) method (Figure S1). Briefly, the theoretical effect of the combination was calculated by adding the effects of each drug used alone at the concentration tested in the combination to that obtained when silibinin was tested alone (i.e., “negative” control plates + effect of silibinin as single agent). This theoretical effect was compared with the actual effect obtained during the combinatorial experiment (“positive” plates, i.e., drugs in combination with silibinin) carried out strictly in parallel. The different interactions were then defined as follows: “additivity” was an observed effect equal to the theoretical effect, and the ratio between them ranged between 0.8 and 1.2; “synergy” was an observed effect higher than the theoretical effect, and the ratio between them was less than 0.8; and “antagonism” was an observed effect lower than the theoretical effect, and the ratio between them was more than 1.2. The interaction between silibinin and a given drug was initially scored as “synergistic” when at least two FEs were <0.8. A truly synergistic interaction was scored when data sets were re-assessed using a stricter threshold criterion (i.e., at least two FEs were <0.6). The representative immunoblots presented in the upper part of the figure show Western blot analyses of cell lysates from A549 parental cells and ALK + A549 derivatives cultured in the absence or presence of graded concentrations of silibinin (24 h) immunoblotted with anti-phospho-STAT3^Tyr705^, anti-total STAT3, and anti-β-actin. Created with BioRender. (+/−, plus/minus).

Although scarce, new studies are beginning to shed light on the ability of silibinin to reverse the multidrug resistance (MDR) phenotype of lung cancer cells. Silibinin has been shown to act synergistically with some chemotherapeutics (e.g., doxorubicin, etoposide) in multidrug-resistant SCLC cells through a mechanism that might involve the direct inhibition of adenosine triphosphate binding cassette (ABC)-transporters such as human P-glycoprotein and multidrug resistance-associated protein-1, as well as the downregulation of the expression of the respective *ABCB1* and *ABCC1* genes [51,52,53,54,55,56]. Because most patients with advanced EGFR- or ALK-positive NSCLC will receive chemotherapy at some point during their treatment course, it would seem desirable to evaluate whether silibinin specifically impacts EGFR mutation- and ALK translocation-driven chemosensitivity profiles. Using the CRISPR/Cas9-edited *EML4-ALK* fusion isogenic model in A549 NSCLC cells, which naturally harbor other genomic aberrations inherent in NSCLC (e.g., *KRAS/STK11* co-mutation), we recently performed a chemical sensitivity screen to evaluate how silibinin modulates the sensitivity of these cells to a variety of chemotherapeutics (Figure 2; Figure S1). The *EML-ALK* fusion CCL-185IG derivative acquired a notably enhanced responsiveness to silibinin when co-treated with the dihydrofolate reductase inhibitor aminopterin––the original clinical anti-folate––and azaserine, a glutamine-fructose-6-phosphate transaminase (GFPT) inhibitor that blocks N-linked glycosylation and the hexosamine biosynthesis pathway. Silibinin co-treatment also prevented EML-ALK fusion-driven resistance to the platinum agents cisplatin and carboplatin. Further studies are warranted to evaluate whether EGFR- and ALK-positive tumors acquire sensitivity to certain silibinin-containing chemotherapeutic combinations once they are resistant to EGFR- and ALK-TKIs and available TKI options are exhausted.

### 5.2. Silibinin and Lung Cancer Metastatic Traits

#### 5.2.1. Inhibition of Cell Invasion

Early studies observed that, in the absence of cytotoxic effects, silibinin could exert dose- and time-dependent inhibitory effects on the invasion and motility (but not on the adhesion) of highly metastatic NSCLC cell models [57]. Mechanistic studies revealed that silibinin decreased the expression of metalloproteinase-2 (MMP-2) and urokinase plasminogen activator, and enhanced the expression of tissue inhibitor of metalloproteinase (TIMP-2) [57]. The negative effect of silibinin on NSCLC invasiveness and metastasis, by changing the balance between MMPs and TIMPs in favor of the inhibitors, appeared to occur downstream of its ability to inactivate PI3K-AKT and MAPK signaling pathways [58,59]. More recent mechanistic studies have established, however, that the mechanism of action of silibinin against MMPs might causally involve silibinin-driven inhibition of STAT3 activation and nuclear translocation [60].

#### 5.2.2. Inhibition of Epithelial-to-Mesenchymal Transition

Beyond MMPs and TIMPs, which have key roles in tumor cell invasion and metastasis by digesting the basement membrane and extracellular matrix components, silibinin can target lung cancer metastastic traits by inhibiting EMT per se. EMT is a highly complex molecular reprogramming process whereby cells lose their epithelial features and acquire a mesenchymal phenotype, allowing them to detach from the primary tumor, invade adjacent stroma, enter systemic circulation, and form distant metastasis. EMT also contributes to tumor aggressiveness by enhancing the resistance of cancer cells to chemotherapy, radiation therapy and targeted therapy, which is a key feature of tumor- and metastasis-initiating CSCs (reviewed in [61,62,63]).

Various mechanisms of resistance to EGFR- and ALK-TKIs in NSCLC are linked to the activation of EMT-like phenomena, irrespective of the EGFR and ALK mutation status [64,65,66,67,68,69,70,71,72]. Silibinin has been reported to restore drug sensitivity to EGFR-mutant NSCLC xenografts with EMT-driven resistance to gefitinib and erlotinib. Silibinin treatment also impedes the regrowth of gefitinib-unresponsive xenograft NSCLC tumors, resulting in drastic tumor growth prevention in vivo [48]. Similarly, silibinin was found to fully activate a reciprocal mesenchymal-to-epithelial transition in erlotinib-refractory cells and prevent the highly migratogenic phenotype of erlotinib-resistant NSCLC cells [49].

The ability of silibinin to block EMT and to impede the acquisition of transcriptional and morphological behavior of transitioning cells appears to occur in a multi-faceted manner. Silibinin can fine-tune the epigenetic dynamics of key EMT-driven events. For instance, silibinin was found to fully reverse the EMT-related high miR-21/low miR-200c microRNA signature and repress the expession of the mesenchymal markers SNAIL, ZEB1, and N-cadherin in erlotinib-refractory NSCLC human xenografts [49]. Because epigenetic modulation of the miR-21 oncogene and the miR-200c tumor suppressor is causally associated with transition to a CSC-like state [73,74,75,76,77], these findings indicated that silibinin might regulate the epigenetic plasticity of microRNAs, contributing to the evolving and adapting phenotypes of lung carcinomas. Indeed, combinatorial treatment with silibinin and histone deacetylase and DNA methyltransferase inhibitors modulated EMT events in NSCLC cell lines, including reversion of the inverse expression pattern of ZEB1 and E-cadherin, tempering their migratory and invasive potential [78]. In the same line, silibinin was recently shown to suppress migration, invasion, and EMT expression by repressing the expression of Rhomboid domain containing 1, a well-known promoter of cell migration, invasion, EMT, and stem cell-like phenotypes in multiple cancer types including lung cancer [79]. The initially reported capacity of silibinin to target EGFR signaling [46] has been shown to involve the suppression of the downstream matrix remodeling enzyme lysyl oxidase, a key contributor to the early steps of metastastic colonization by enhancing tumor invasion, migration, and the formation of pre-metastatic niche [80,81,82,83]. Silibinin in combination with EGFR blockade prevented NSCLC cell migration in vitro and tumor metastasis in an orthotopic implantation metastasis model by targeting the EGFR/LOX pathway [84]. In contrast to other EMT-targeting compounds, a recent transcriptomic profiling study revealed that de novo responsiveness of NSCLC cells to silibinin does not correlate with their intrinsic EMT stage [85]. Rather, silibinin responsiveness appears to be linked to a subnetwork of tightly interconnected genes of cell cycle, survival, and stress response (e.g., *BIRC5*, *FOXM1*, and *BRCA1*) whose transcriptomic pattern is under control of STAT3 [85].

#### 5.2.3. Inhibition of Brain Metastasis

Our resent findings have positioned silibinin as a successful therapy to treat established brain metastasis in patients with NSCLC. In 2016, we presented the first evidence for oral silibinin as part of a bioavailable formulation with predicted capacity to cross the blood–brain barrier (BBB) [86], which resulted in significant clinical and radiological improvement of brain metastasis in two patients with poor performance status that progressed after whole brain radiotherapy and chemotherapy [87]. The suppressive effects of silibinin on progressive brain metastasis, which included a marked reduction in peritumoral brain edema, occurred in the absence of changes to the primary lung tumor outgrowth [87]. We then compared our clinical series of patients with NSCLC treated with the silibinin-containing nutraceutical Legasil^®^ (*n* = 18; single-agent silibinin *n* = 3 and silibinin plus additional therapy *n* = 15) with patients treated at the same institution who completed whole-brain radiation therapy for NSCLC brain metastasis and who received systemic therapy but not silibinin (*n* = 38). In such a small cohort, silibinin demonstrated highly significant clinical activity with a 75% overall response rate in the brain including three complete responses and ten partial responses [88]. Indeed, the patients receiving silibinin as palliative care (*n* = 3) benefited from additional treatment lines as a result of their general status improvement and magnetic resonance imaging-based brain responses. The overall survival from the diagnosis of brain metastasis was significantly superior in the cohort of patients treated with the silibinin-containing nutraceutical (15.5 months) than in the control cohort (4.0 months), a trend that was maintained when patients with EGFR and ALK oncogenic driver mutations were excluded from the analysis [88].

A subpopulation of reactive astrocytes surrounding brain metastases has been identified that is driven by STAT3 activation and is characterized by nuclear accumulation of phospho-active STAT3 [88,89]. NSCLC metastatic tumor cells that have initiated a brain macro-metastasis secrete various factors that trigger astrocytes in the surrounding area to become reactive with enhanced STAT3 activation. In turn, phospho-STAT3+ reactive astrocytes produce cytokines and other factors to escape innate and adaptive anti-tumor immune responses [88]. Investigations into the molecular mechanisms involved in the aforementioned clinico-molecular activities of silibinin revealed that silibinin efficiently suppresses the ability of brain metastastic NSCLC cells to co-opt a pro-metastatic program driven by STAT3 in a subpopulation of reactive astrocytes surrounding metastatic lesions [88]. Blocking STAT3 signaling in reactive astrocytes in the brain microenvironment with silibinin reduced brain metastasis growth and disease burden.

## 6. STAT3: A primary Tumor-Cell Intrinsic and Microenvironmental Target of Silibinin in Lung Cancer

**Figure 3:**
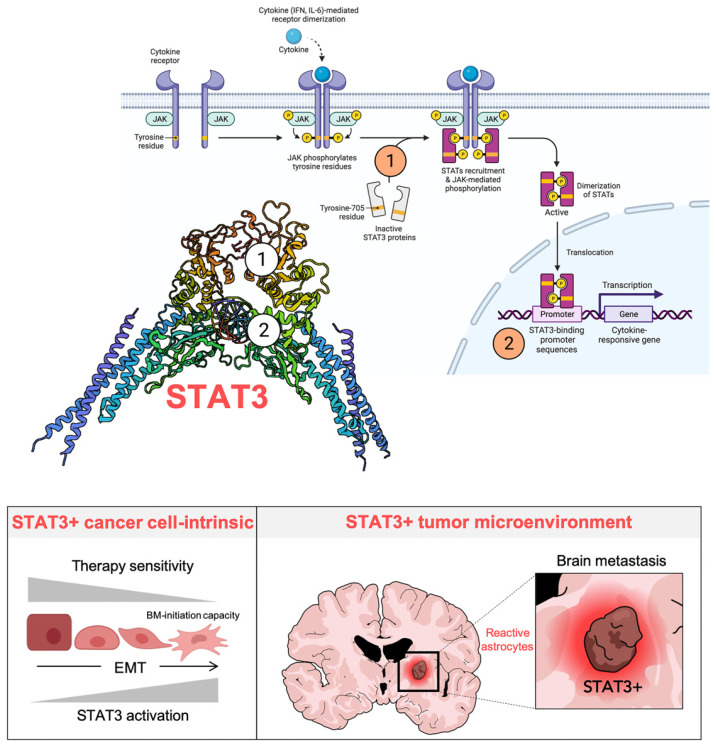
Silibinin mechanism of action in lung cancer: A STAT3-centric view. Aberrant activation of JAK/STAT3 signaling, in particular STAT3, participates in the initiation, development, and therapeutic resistance of lung cancer via promotion of proliferation, survival, inflammation, angiogenesis, and metastasis. Silibinin is a unique blocker of the JAK/STAT3 signal transduction cascade that operates as a bimodal SH2- and DBD-targeting direct STAT3 inhibitor (STAT3i) while sparing JAK activity. STAT3 participates in multiple layers of the EMT regulatory network, and feedback activation of STAT3 is a common cause of resistance to many chemotherapies and targeted cancer therapies. At the lung cancer cell-intrinsic level, silibinin-containing combinatorial treatments can overcome drug resistance and reduce the brain metastasis-initiating capacity of lung cancer cells. Brain metastasis cells promote the co-option of a pro-metastatic program driven by STAT3 activation in a subpopulation of reactive astrocytes surrounding metastatic lesions. Blocking microenvironmental STAT3 signaling in reactive astrocytes with silibinin reduces the growth of brain metastases from primary NSCLC tumors, even at advanced stages of colonization. Created with BioRender.

Central to the tumor cell-intrinsic and microenvironmental effects of silibinin in lung cancer is the transcriptional factor STAT3 (Figure 3).

### 6.1. Identification of Silibinin as a Direct STAT3 Inhibitor

We recently combined experimental, computational, and clinical efforts to investigate how silibinin imparts therapeutic benefits to patients with lung cancer by targeting STAT3. We found that the primary mechanism of action of silibinin involves a unique, bimodal Src Homology-2 domain (SH2; STAT3 dimerization) and DBD (STAT3 DNA-binding domain)-targeted inhibitory effect against STAT3 [89]. Biochemical approaches demonstrated that silibinin attenuates the tyrosine (Y705) phospho-activation in GFP-STAT3 genetic fusions without significantly altering the kinase activity of the STAT3 upstream kinases JAK1 and JAK2. Once we discarded the possibility that silibinin was a direct JAK inhibitor, we performed a comparative computational study based on docking and molecular dynamics simulations over structurally diverse STAT3 inhibitors. Silibinin was predicted to show a unique mode of high-affinity binding to the SH2 domain, partially overlapping with the cavity occupied by other direct STAT3 inhibitors to indirectly prevent Y705 phosphorylation. Silibinin treatment of cultured NSCLC cells prevented IL-6 inducible, constitutive, and acquired feedback activation of STAT3 [89]. In silico approaches also predicted that silibinin could directly bind with high affinity to the STAT3 DBD, uniquely involving the establishment of direct interactions with DNA. Because STAT3 dimerization is mediated by the interaction between a phospho-Y705-containing peptide and the SH2 domain, which is essential for its DNA binding and subsequent transcriptional activity, the demonstration that silibinin prevented STAT3 nuclear translocation, blocked the binding of activated STAT3 to its consensus DNA sequence, and suppressed STAT3-directed transcriptional activity further confirmed the molecular behavior of silibinin as a bona fide direct STAT3 inhibitor [89].

### 6.2. STAT3-Targeted Cancer Cell-Intrinsic and Microenvironmental Effects of Silibinin

The so-called STAT3C mutant, a constitutively active form of STAT3, has been employed to confirm STAT3 as a primary tumor-cell intrinsic and microenvironmental target of silibinin [90,91]. This mutant has substitutions of the A661 and N663 residues of the SH2 domain with cysteines, allowing a disulfide bond to form between two unphosphorylated STAT3 monomers; yet, it still requires Y705 phosphorylation for functional activation via promotion of maximal DNA binding affinity and protection from inactivation by phosphatases (slower off-rate), resulting in the accumulation of transcriptionally active STAT3 dimer complexes. In silico modeling of the conformation of silibinin in the binding pocket within the SH2 domain of native and A662C/N664C-mutant structures predicted a reduced ability of silibinin to bind with high affinity to the SH2 domain of the STAT3C mutant [88]. Accordingly, cancer cells engineered to overexpress STAT3C remain largely unresponsive to the inhibitory effects of silibinin on key transcriptional and phenotypic targets of STAT3 (e.g., c-myc expression and metabolic reprogramming) [88,92]. Moreover, overexpression of constitutively active STAT3C in astrocytes suffices to prevent the regulatory effects of silibinin, thus demonstrating the STAT3-dependency on the phenotypic effects of silibinin towards the microenvironment of NSCLC brain metastasis [88].

We should acknowledge that STAT3 might also represent a potential therapeutic target in the early prevention/treatment of lung-to-brain metastases. Using patient-derived stem cell lines from lung-to-brain metastases, Singh and colleagues identified STAT3 and miR-21 as cooperative regulators of stemness, migration, and brain-metastasis initiation capacity of lung cancer cells [93]. The dual STAT3/miR-21 inhibitory activity of silibinin [49,89] might therefore be revisited in terms of its ability to target not only the growth of established brain metastasis, but also the early machinery activated by brain-metastasis initiating cells to escape the primary lung tumor, migrate, and invade the neural niche.

**Figure 4:**
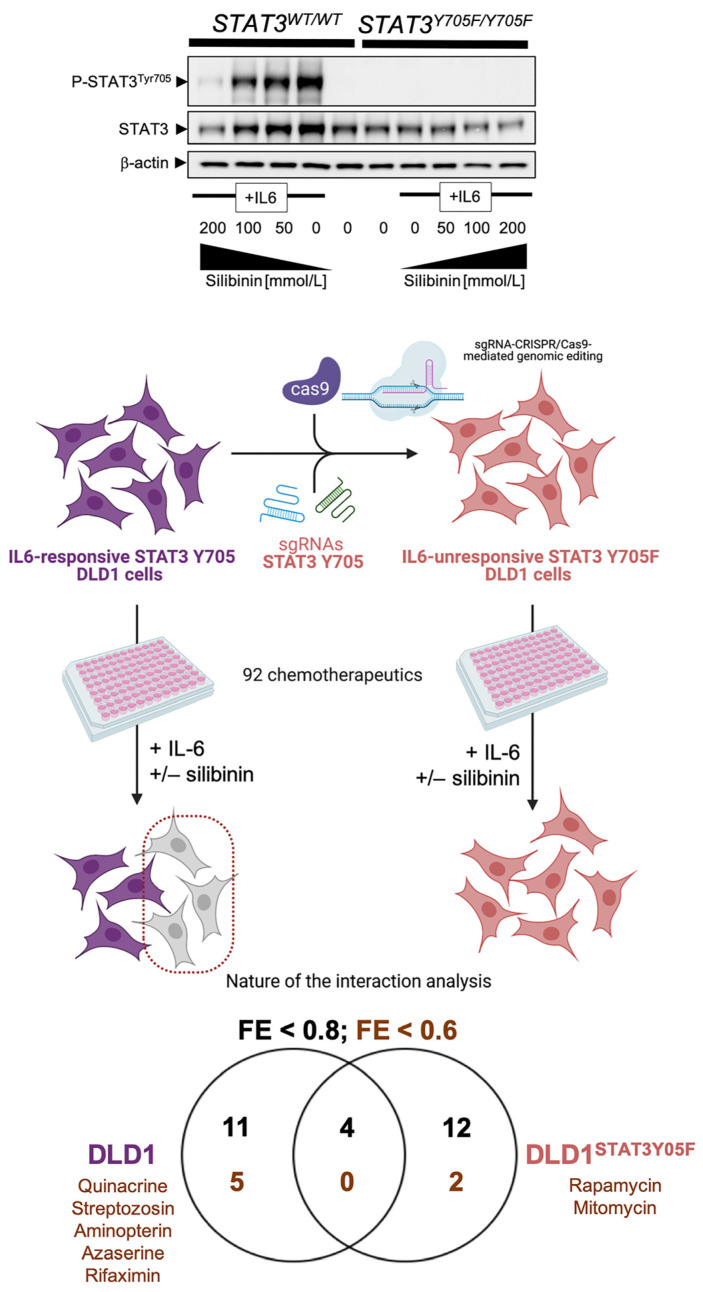
STAT3 Tyr705-dependent chemosensitizing effects of silibinin. We utilized the Phenotypic Microarray system, marketed and sold by Biolog (www.biolog.com, access date: 30 May 2021), to measure the sensitivity of DLD1 (STAT3^WT/WT^) cancer cells and a homozygous STAT3^Y705F/Y705F^ knock-in isogenic derivative (Horizon Discovery, Cat.# HD 115-016) to a wide variety of 92 growth inhibitors in microplates (PM-M11 to PM-M14) following an identical procedure to that described in Figure 2. The representative immunoblots presented in the upper part of the figure show western blot analyses of cell lysates from DLD1 STAT3*^WT^*^/*WT*^ parental cells and DLD1 STAT3^Y705F/Y705F^ derivatives cultured in the absence or presence of graded concentrations of silibinin (24 h) immunoblotted with anti-phospho-STAT3^Tyr705^, anti-total STAT3, and anti-β-actin. Created with BioRender. (+/−, plus/minus).

Taking advantage of the CRISPR/Cas9-edited homozygous Y705F mutant STAT3 protein in DLD-1^STAT3Y705F/Y705F^ cells, we recently performed a chemical sensitivity screen to evaluate how STAT3 phosphorylation at Tyr705 might be required for silibinin-induced chemosensitization events (Figure 4; Figure S2). The ability of silibinin to synergistically cooperate with aminopterin was lost in DLD-1^STAT3Y705F/^^Y705F^ cells, thereby suggesting that the nature of the interaction more likely relied on the capacity of aminopterin to operate as a JAK/STAT inhibitor independently of its primary dihydrofolate reductase target [94]. The synergistic interaction between silibinin and the GFPT inhibitor azaserine was, however, only partially prevented when the ability of silibinin to block IL6-induced Y705 phosphorylation was abolished, suggesting that silibinin may directly operate on the N-linked glycosylation/hexosamine biosynthesis pathway.

### 6.3. Silibinin versus Other Natural Products Exhibiting STAT3 Inhibitory Activity

Natural products have historically been an important resource of chemical scaffolds and bioactive substructures in the discovery of STAT3 inhibitors. A large list of natural products have been reported in the literature to exhibit STAT3 inhibitory activity, including curcumin, berbamine, resveratrol, caffeic acid, capsaicin, cryptotanshinone, celastrol, avicin D, withaferin A, betulinic acid, ursolic acid, oleanolic acid, cucurbitacin, diosgenin, emodin, honokiol, flavopiridol, evodiamine, carbazole, sanguarine, and guggulsterone (reviewed in [95]). Despite the fact some of these natural products have reached clinical development, the precise STAT3-targeting mechanism(s) of action of the majority has yet to be fully elucidated, as they might inhibit STAT3 indirectly and are expected to block several targets. Resveratrol (3,5,4′-trihydroxystilbene), a widely studied polyphenolic compound found in red grapes and several other plants, was originally reported to inhibit constitutive and IL-6-induced STAT3 activity in multiple tumor cell types [96,97]. Although thought to be primarily a STAT3 inhibitor, resveratrol has also been found to modulate STAT1 activity, thus highlighting that selectivity for STAT3 over STAT1 should be carefully considered for the development of natural product-like STAT3 inhibitors [98]. Comparative in silico docking studies aimed to study the binding specificity of STAT inhibitors established that those compounds exclusively targeting the highly conserved phosphotyrosine binding pocket of the SH2 domain should be expected to lack selectivity towards STAT3, given that STAT1 and STAT3 have identical active residues at this site [99,100]. The predicted ability of silibinin to bind the SH2 activation/dimerization domain relies on its capacity to overlap with up to 60% of all the residues involved in the binding mode of a wide variety of structurally diverse STAT3is, but showing a unique binding mode [89]. By targeting the SH2 domain of STAT3 monomers, silibinin can prevent not only binding of STAT3 to activated cell surface receptors, but also to block cytosolic STAT3 dimerization, thereby preventing nuclear accumulation of phospho-active STAT3 [89]. Importantly, the ability of silibinin to inhibit the transcriptional activity of STAT3 in cells does not rely exclusively on its ability to antagonize STAT3 dimerization in the cytosol and STAT3 tyrosine phosphorylation, but also involves an additional direct inhibition of STAT3 via binding to the DBD irrespective of the STAT3 dimerization status [89]. Accordingly, silibinin is the best-positioned natural lead for a new generation of bimodal SH2- and DBD-targeting STAT3is that might become incorporated into the clinical management of lung tumors. While the clinical value of silibinin as a bona fide anti-lung cancer therapy remains uncertain with respect to its bioavailability and BBB permeability, we are rapidly accumulating information to help identify the best silibinin formulation that would reach cancer tissues and have clinical activity, including a meaningful formulation against lung brain metastases [86].

## 7. Silibinin and Lung Cancer: The Past, Present, and Future (a Corollary)

The milk thistle, whose main bioactive component is the flavonolignan silibinin, was originally described as a remedy for the bites of poisonous snakes in “*De Materia Medica*” by Dioscorides (50 A.D.). Almost 2000 years later, new formulations of silibinin are being clinically developed to protect liver against injury from mushroom poisoning or lipotoxic injury in fatty liver diseases. An ever-expanding number of studies are exploring the capacity of silibinin to exert inhibitory activity against cultured cancer cells and tumor xenografts, to enhance the efficacy of other therapeutic agents, and to overcome the emergence of cancer drug resistance in pre-clinical lung cancer models [101]. Although silibinin has shown chemopreventive and chemosensitizing activity against various human malignancies through multiple molecular pathways [102,103], lung cancer is becoming the paradigm for how the deconstruction of a central mechanism of action of silibinin (i.e., STAT3) has enabled this natural compound to reach clinical development. Perhaps more importantly, silibinin-driven STAT3 blockade holds immense promise in areas of highly unmet clinical need such as lung cancer brain metastasis, which portend a poor prognosis and have very few therapeutic options [87,88]. Here, we have reviewed the historical context and provided new translational insights into how an old hepatoprotective remedy could be viewed as a novel lung cancer-preventive and therapeutic biomolecule, which might serve as a guiding example for other tumor types in the future.

Forthcoming studies should accept the challenge of developing silibinin and/or next-generation silibinin derivatives with improved lung cancer-preventing and treatment traits. We need to disentangle how silibinin prevents the generation of metastasis-initiating subpopulations within chemoresistant and/or TKI-tolerant lung tumors. In this regard, it would be important to elucidate the molecular mechanisms through which silibinin prevents brain tropism of metastatic lung cancer cells by targeting their capacities to self-renew and/or remodel the tumor microenvironment. We also need to molecularly deconstruct and functionally monitor the ability of silibinin to regulate the immune-escape mechanisms of lung cancer cells (and/or brain metastasis-initiating lung cancer cells), to influence the response to T-cells, and to interact with immune checkpoint inhibitors (e.g., anti-CTLA-4 and anti-PD-1/PD-L1 antibodies) in therapy-resistant lung carcinomas. Finally, we need to evaluate how silibinin interacts with the BBB to impede transmigration of brain metastasis-initiating cells and/or to regulate the metabolism and brain accumulation of targeted therapies. The unraveling of an unforeseen, selective vulnerability of silibinin-treated tumor cells to the hexosamine biosynthesis pathway inhibitor azaserine using genomically edited isogenic models might exemplify how to exploit the therapeutic usage of silibinin in combination with certain targetable weaknesses in specific subtypes of lung cancer (e.g., *KRAS/STK11* co-mutant tumors with dependence on the hexosamine biosynthesis pathway through GFPT2 [104]). Using silibinin as a lead structure to guide development, it would be possible to use synthetic chemistry approaches to generate a battery of silibinin derivatives with enhanced radiosensitizing capacity and augmented brain targeting. These approaches, together with the utilization of clinically relevant models of lung cancer to test the efficacy and toxicity of silibinin and/or silibinin derivatives, should allow for the incorporation of this flavonolignan as a modern therapeutic approach for medical management of human lung cancer.

## 8. Conclusions

- The deconstruction and validation of a central mechanism of action of silibinin (i.e., STAT3) has enabled this natural compound to reach clinical development in lung cancer;
- Silibinin is capable of reaching target cancer tissues and groundbreakingly provides survival advantages to lung cancer patients with brain metastasis when used as part of formulations with an optimized oral bioavailability;
- Critical drivers for silibinin responsiveness versus resistance in specific lung cancer molecular subtypes can be identified using CRISPR-based functional genomics;
- Lessons from natural chemistry of silibinin can offer novel approaches for synthetic chemistry in lung cancer drug discovery.

## Data Availability

The data that support the findings of this study are available from the corresponding authors, upon reasonable request.

## Supplementary Materials

The following are available online at https://www.mdpi.com/article/10.3390/ph14060559/s1, Figure S1. (a) Original uncropped immunoblots for Figure 2. (b) Original raw data of the phenotypic microarray system analyzed in Figure 2. Figure S2. (a) Original uncropped immunoblots for Figure 4. (b) Original raw data of the phenotypic microarray system analyzed in Figure 4.
