# Supplementary material for: Lung Cancer Management with Silibinin: A Historical and Translational Perspective
Source: Pharmaceuticals (Basel). 2021 Jun 11;14(6):559. doi: 10.3390/ph14060559 (PMC8230811; doi:10.3390/ph14060559)
Supplement: Supplementary file 1 [file pharmaceuticals-14-00559-s001.zip › pharmaceuticals-1237764-supplementary.pdf]

Figure S1. (**a**) Original uncropped immunoblots for Figure 2. (**b**) Original raw data of the phenotypic microarray system analyzed in Figure 2.

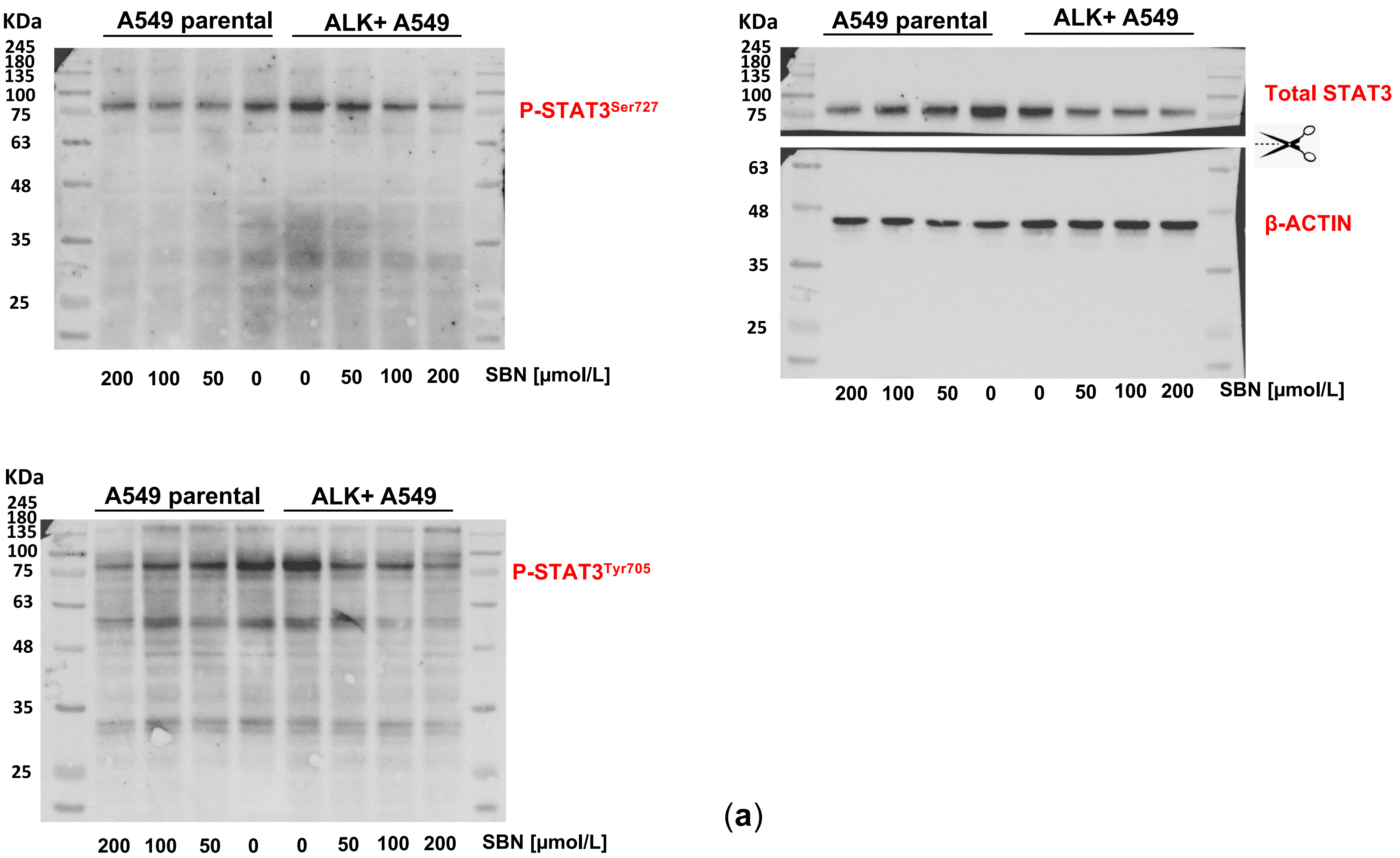

(**a**)

(b)

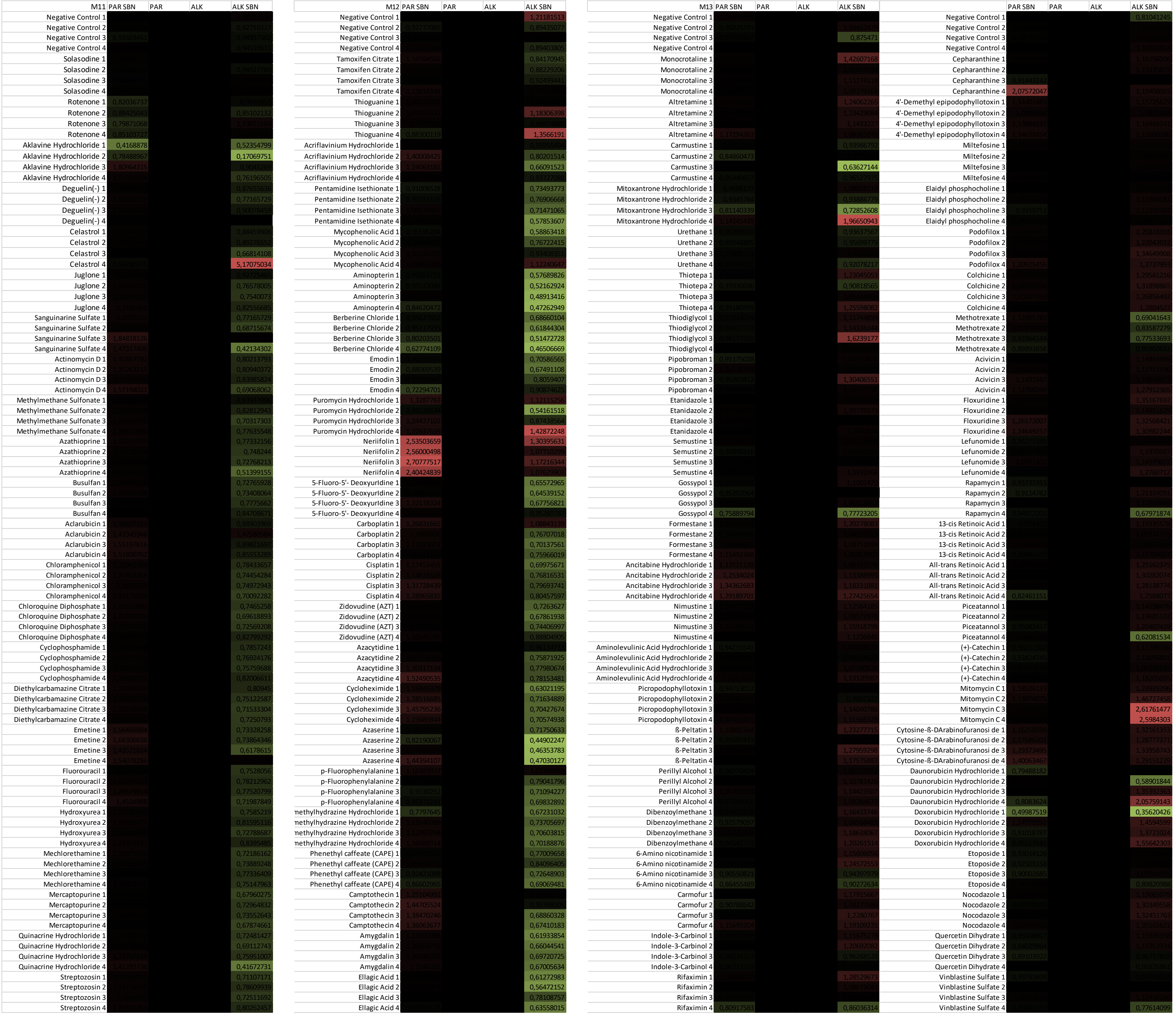
M11 PAR SBN PAR ALK ALK SBN
M12 PAR SBN PAR ALK ALK SBN
M13 PAR SBN PAR ALK ALK SBN
PAR SBN PAR ALK ALK SBN

Figure S2. (**a**) Original uncropped immunoblots for Figure 4. (**b**) Original raw data of the phenotypic microarray system analyzed in Figure 4.

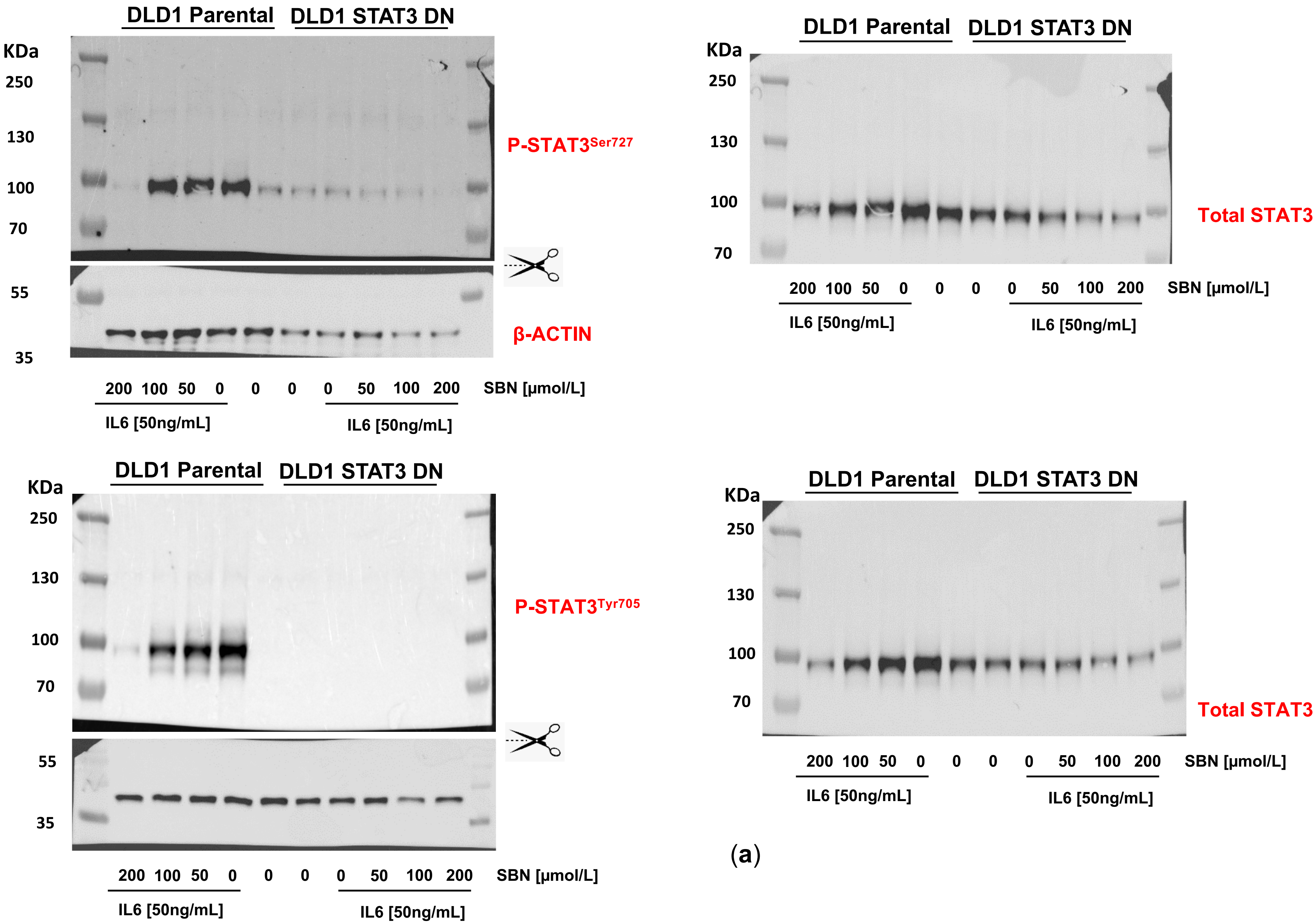

(**a**)

(b)

M11	PAR SBN	PAR	DN	DN SBN
Negative Control 1				
Negative Control 2				
Negative Control 3	[illegible]			
Negative Control 4	[illegible]			[illegible]
Solasodine 1				
Solasodine 2				[illegible]
Solasodine 3				
Solasodine 4				[illegible]
Rotenone 1				[illegible]
Rotenone 2				0,71748669
Rotenone 3				[illegible]
Rotenone 4	0,77787316			[illegible]
Aklavine Hydrochloride 1				[illegible]
Aklavine Hydrochloride 2				0,57535014
Aklavine Hydrochloride 3	[illegible]			[illegible]
Aklavine Hydrochloride 4				
Deguelin(-) 1	[illegible]			0,80663795
Deguelin(-) 2				
Deguelin(-) 3				
Deguelin(-) 4				
Celastrol 1	[illegible]			
Celastrol 2				[illegible]
Celastrol 3				[illegible]
Celastrol 4				34,4833089
Juglone 1				0,76831275
Juglone 2	[illegible]			0,80375226
Juglone 3	[illegible]			
Juglone 4	[illegible]			0,84895416
Sanguinarine Sulfate 1				
Sanguinarine Sulfate 2				[illegible]
Sanguinarine Sulfate 3	0,81578416			
Sanguinarine Sulfate 4	0,81462247			
Actinomycin D 1				[illegible]
Actinomycin D 2				[illegible]
Actinomycin D 3				[illegible]
Actinomycin D 4				
Methylmethane Sulfonate 1	0,58355689			[illegible]
Methylmethane Sulfonate 2				[illegible]
Methylmethane Sulfonate 3	[illegible]			
Methylmethane Sulfonate 4	0,85849195			0,85553905
Azathioprine 1	[illegible]			0,83047101
Azathioprine 2	[illegible]			
Azathioprine 3				[illegible]
Azathioprine 4	0,49222302			[illegible]
Busulfan 1	[illegible]			
Busulfan 2	[illegible]			[illegible]
Busulfan 3	[illegible]			[illegible]
Busulfan 4	0,68885159			0,83803592
Aclarubicin 1	0,61789766			0,57195712
Aclarubicin 2	[illegible]			0,66875581
Aclarubicin 3				
Aclarubicin 4				
Chloramphenicol 1				
Chloramphenicol 2	0,88910417			0,82437528
Chloramphenicol 3	0,77716262			[illegible]
Chloramphenicol 4	0,75085326			[illegible]
Chloroquine Diphosphate 1	[illegible]			
Chloroquine Diphosphate 2				[illegible]
Chloroquine Diphosphate 3				
Chloroquine Diphosphate 4	0,77648217			[illegible]
Cyclophosphamide 1	0,64322603			[illegible]
Cyclophosphamide 2				[illegible]
Cyclophosphamide 3	[illegible]			
Cyclophosphamide 4	[illegible]			
Diethylcarbamazine Citrate 1	[illegible]			0,87069844
Diethylcarbamazine Citrate 2	[illegible]			
Diethylcarbamazine Citrate 3	[illegible]			
Diethylcarbamazine Citrate 4	0,85350754			[illegible]
Emetine 1				
Emetine 2	[illegible]			
Emetine 3				
Emetine 4				
Fluorouracil 1				0,75313913
Fluorouracil 2				0,81418828
Fluorouracil 3				[illegible]
Fluorouracil 4				
Hydroxyurea 1				0,81074285
Hydroxyurea 2	[illegible]			0,86917477
Hydroxyurea 3	0,72893593			0,8456255
Hydroxyurea 4	[illegible]			
Mechlorethamine 1				
Mechlorethamine 2	[illegible]			0,83556394
Mechlorethamine 3	0,82788664			[illegible]
Mechlorethamine 4	0,75177385			0,82715269
Mercaptopurine 1				[illegible]
Mercaptopurine 2	0,82066431			0,82573852
Mercaptopurine 3				
Mercaptopurine 4	[illegible]			
Quinacrine Hydrochloride 1	0,27124744			
Quinacrine Hydrochloride 2				0,86300247
Quinacrine Hydrochloride 3	0,57473454			[illegible]
Quinacrine Hydrochloride 4	0,72403881			0,16195731
Streptozosin 1	0,84992785			0,83699555
Streptozosin 2				
Streptozosin 3	0,37320098			
Streptozosin 4	0,49493967			0,83632106

M12	PAR SBN	PAR	DN	DN SBN
Negative Control 1				[illegible]
Negative Control 2				[illegible]
Negative Control 3	0,89863464			
Negative Control 4				
Tamoxifen Citrate 1				
Tamoxifen Citrate 2	0,82229311			[illegible]
Tamoxifen Citrate 3	[illegible]			
Tamoxifen Citrate 4				[illegible]
Thioguanine 1	[illegible]			
Thioguanine 2				[illegible]
Thioguanine 3				
Thioguanine 4				[illegible]
Acriflavinium Hydrochloride 1	0,80453137			
Acriflavinium Hydrochloride 2	0,80713489			0,74016196
Acriflavinium Hydrochloride 3	0,6703503			0,46217494
Acriflavinium Hydrochloride 4				0,84760781
Pentamidine Isethionate 1	0,87964879			[illegible]
Pentamidine Isethionate 2	0,6510809			0,6094316
Pentamidine Isethionate 3	0,92595928			0,7830841
Pentamidine Isethionate 4	0,93082766			0,8780743
Mycophenolic Acid 1	0,78864616			0,85628665
Mycophenolic Acid 2	0,80299061			
Mycophenolic Acid 3				[illegible]
Mycophenolic Acid 4	[illegible]			
Aminopterin 1	0,65329456			[illegible]
Aminopterin 2	0,6664785			
Aminopterin 3	0,61693849			
Aminopterin 4	0,66087915			
Berberine Chloride 1	0,87043061			0,83944603
Berberine Chloride 2	0,6521924			
Berberine Chloride 3	[illegible]			0,73799321
Berberine Chloride 4	0,54628977			0,69852694
Emodin 1	0,85881699			0,87783734
Emodin 2	0,92581097			0,82762317
Emodin 3	0,8588682			[illegible]
Emodin 4				
Puromycin Hydrochloride 1	[illegible]			[illegible]
Puromycin Hydrochloride 2				[illegible]
Puromycin Hydrochloride 3				[illegible]
Puromycin Hydrochloride 4	[illegible]			[illegible]
Neriifolin 1	[illegible]			
Neriifolin 2	[illegible]			0,80429359
Neriifolin 3	[illegible]			
Neriifolin 4	[illegible]			0,77479478
5-Fluoro-5'- Deoxyuridine 1	0,80715692			0,79907218
5-Fluoro-5'- Deoxyuridine 2	0,83710923			0,77733675
5-Fluoro-5'- Deoxyuridine 3	[illegible]			0,75792573
5-Fluoro-5'- Deoxyuridine 4				
Carboplatin 1	0,92076379			
Carboplatin 2	0,8914883			0,78969208
Carboplatin 3	0,79142096			0,90520866
Carboplatin 4	0,71065217			0,74573077
Cisplatin 1	0,66234366			
Cisplatin 2	0,8596121			0,8823705
Cisplatin 3	0,83099589			0,77581604
Cisplatin 4	0,56792438			0,84335291
Zidovudine (AZT) 1	0,67309867			0,93319474
Zidovudine (AZT) 2	0,69199839			0,75930391
Zidovudine (AZT) 3	0,78324959			
Zidovudine (AZT) 4				
Azacytidine 1	0,73472106			
Azacytidine 2	0,68505878			
Azacytidine 3	0,91819397			
Azacytidine 4				
Cycloheximide 1	0,80707988			0,58978342
Cycloheximide 2				0,75377472
Cycloheximide 3	[illegible]			0,8470672
Cycloheximide 4	0,887556			[illegible]
Azaserine 1	0,73979435			0,75457585
Azaserine 2	0,6031667			0,83137968
Azaserine 3	0,63691803			0,7537428
Azaserine 4	0,48678886			0,841129
p-Fluorophenylalanine 1	0,87395763			0,93855061
p-Fluorophenylalanine 2	0,88495584			0,88642802
p-Fluorophenylalanine 3				0,78116599
p-Fluorophenylalanine 4				0,83096755
nethylhydrazine Hydrochloride 1				0,81298838
nethylhydrazine Hydrochloride 2	[illegible]			0,91518831
nethylhydrazine Hydrochloride 3	[illegible]			0,77106828
nethylhydrazine Hydrochloride 4				0,84348476
Phenethyl caffeate (CAPE) 1				0,92521261
Phenethyl caffeate (CAPE) 2	0,79989267			0,75366915
Phenethyl caffeate (CAPE) 3	0,88298426			0,88126246
Phenethyl caffeate (CAPE) 4	[illegible]			[illegible]
Camptothecin 1	[illegible]			
Camptothecin 2				0,72548049
Camptothecin 3				[illegible]
Camptothecin 4	[illegible]			
Amygdalin 1	[illegible]			0,80094301
Amygdalin 2				0,5362082
Amygdalin 3	4,40664383			0,82431379
Amygdalin 4				
Ellagic Acid 1	0,67363968			0,66591932
Ellagic Acid 2	0,89693638			
Ellagic Acid 3				[illegible]
Ellagic Acid 4	[illegible]			0,87777706

M13	PAR SBN	PAR	DN	DN SBN
Negative Control 1				[illegible]
Negative Control 2				[illegible]
Negative Control 3	[illegible]			[illegible]
Negative Control 4	[illegible]			
Monocrotaline 1				0,81768293
Monocrotaline 2	0,88501963			0,77536292
Monocrotaline 3				[illegible]
Monocrotaline 4				[illegible]
Altretamine 1				0,81150411
Altretamine 2	0,78761619			[illegible]
Altretamine 3				
Altretamine 4				[illegible]
Carmustine 1	[illegible]			[illegible]
Carmustine 2	[illegible]			
Carmustine 3	[illegible]			
Carmustine 4	[illegible]			0,90829706
Mitoxantrone Hydrochloride 1				[illegible]
Mitoxantrone Hydrochloride 2				
Mitoxantrone Hydrochloride 3				0,76403797
Mitoxantrone Hydrochloride 4	0,67207486			
Urethane 1				0,83633918
Urethane 2	0,90752232			
Urethane 3				
Urethane 4	[illegible]			
Thiotepa 1				
Thiotepa 2				[illegible]
Thiotepa 3	[illegible]			
Thiotepa 4	[illegible]			[illegible]
Thiodiglycol 1	[illegible]			
Thiodiglycol 2	[illegible]			
Thiodiglycol 3				
Thiodiglycol 4	[illegible]			[illegible]
Pipobroman 1				
Pipobroman 2	[illegible]			
Pipobroman 3	0,93785416			[illegible]
Pipobroman 4	0,87790058			
Etanidazole 1	0,83775453			
Etanidazole 2	[illegible]			0,88244754
Etanidazole 3	0,91626281			0,89197840
Etanidazole 4	0,78719792			
Semustine 1				
Semustine 2	0,88219125			[illegible]
Semustine 3	0,87259137			[illegible]
Semustine 4	0,87455337			
Gossypol 1	0,85558474			0,88400812
Gossypol 2	0,88109535			0,88337150
Gossypol 3	0,85768608			
Gossypol 4	0,71302566			[illegible]
Formestane 1	0,87130652			0,85588065
Formestane 2	0,8360696			
Formestane 3	[illegible]			
Formestane 4	[illegible]			
Ancitabine Hydrochloride 1				[illegible]
Ancitabine Hydrochloride 2	[illegible]			
Ancitabine Hydrochloride 3				[illegible]
Ancitabine Hydrochloride 4				[illegible]
Nimustine 1	0,86690008			
Nimustine 2	0,87096681			
Nimustine 3	[illegible]			
Nimustine 4	0,63367413			
Aminolevulinic Acid Hydrochloride 1	1,5827282			
Aminolevulinic Acid Hydrochloride 2				[illegible]
Aminolevulinic Acid Hydrochloride 3	[illegible]			
Aminolevulinic Acid Hydrochloride 4	0,75788906			[illegible]
Picropodophyllotoxin 1	0,88132523			
Picropodophyllotoxin 2				[illegible]
Picropodophyllotoxin 3				[illegible]
Picropodophyllotoxin 4	[illegible]			[illegible]
ß-Peltatin 1	[illegible]			[illegible]
ß-Peltatin 2	[illegible]			[illegible]
ß-Peltatin 3				[illegible]
ß-Peltatin 4	0,83327394			[illegible]
Perillyl Alcohol 1	1,32206111			[illegible]
Perillyl Alcohol 2	0,92156711			0,91006548
Perillyl Alcohol 3				
Perillyl Alcohol 4	0,59263941			0,81517346
Dibenzoylmethane 1	[illegible]			[illegible]
Dibenzoylmethane 2				0,84688477
Dibenzoylmethane 3	0,88675445			[illegible]
Dibenzoylmethane 4				
6-Amino nicotinamide 1				0,8158379
6-Amino nicotinamide 2	0,7960069			0,87082988
6-Amino nicotinamide 3	0,68841089			0,71091644
6-Amino nicotinamide 4	0,4537891			[illegible]
Carmofur 1	2,07442832			
Carmofur 2	[illegible]			0,78963974
Carmofur 3				[illegible]
Carmofur 4	0,20859826			
Indole-3-Carbinol 1	[illegible]			[illegible]
Indole-3-Carbinol 2	0,39213437			0,87718267
Indole-3-Carbinol 3	0,80718342			[illegible]
Indole-3-Carbinol 4	0,88857733			
Rifaximin 1	0,85257015			1,71347044
Rifaximin 2	0,73544279			[illegible]
Rifaximin 3	0,47769103			[illegible]
Rifaximin 4	0,28729259			[illegible]

M14	PAR SBN	PAR	DN	DN SBN
Negative Control 1				
Negative Control 2	[illegible]			[illegible]
Negative Control 3	[illegible]			[illegible]
Negative Control 4				[illegible]
Cepharanthine 1				
Cepharanthine 2	[illegible]			[illegible]
Cepharanthine 3				
Cepharanthine 4	[illegible]			0,76549239
4'-Demethyl epipodophyllotoxin 1				[illegible]
4'-Demethyl epipodophyllotoxin 2				[illegible]
4'-Demethyl epipodophyllotoxin 3				
4'-Demethyl epipodophyllotoxin 4				
Miltefosine 1				
Miltefosine 2	[illegible]			
Miltefosine 3				
Miltefosine 4	[illegible]			[illegible]
Elaidyl phosphocholine 1	0,87671723			0,9078538
Elaidyl phosphocholine 2				0,84656941
Elaidyl phosphocholine 3	0,90264457			0,89089905
Elaidyl phosphocholine 4	0,92448703			0,82124745
Podofilox 1	[illegible]			
Podofilox 2				
Podofilox 3	[illegible]			
Podofilox 4				
Colchicine 1				[illegible]
Colchicine 2				[illegible]
Colchicine 3	[illegible]			[illegible]
Colchicine 4	[illegible]			[illegible]
Methotrexate 1	0,89202953			[illegible]
Methotrexate 2	[illegible]			[illegible]
Methotrexate 3	0,88682626			[illegible]
Methotrexate 4	0,88029106			[illegible]
Acivicin 1				0,80716215
Acivicin 2	0,77153047			0,63976899
Acivicin 3				0,86691643
Acivicin 4	0,87858763			0,62456582
Floxuridine 1				
Floxuridine 2	[illegible]			[illegible]
Floxuridine 3	[illegible]			[illegible]
Floxuridine 4	[illegible]			[illegible]
Lefunomide 1				0,73258158
Lefunomide 2				0,70452396
Lefunomide 3				0,77384855
Lefunomide 4	[illegible]			0,64844549
Rapamycin 1				0,60918901
Rapamycin 2	0,86726209			0,63992583
Rapamycin 3	0,8188293			0,69834001
Rapamycin 4	0,44357619			0,57478047
13-cis Retinoic Acid 1				0,86254995
13-cis Retinoic Acid 2				0,81220684
13-cis Retinoic Acid 3				
13-cis Retinoic Acid 4				0,88148448
All-trans Retinoic Acid 1				0,85437591
All-trans Retinoic Acid 2	[illegible]			0,8373899
All-trans Retinoic Acid 3				0,92178077
All-trans Retinoic Acid 4				
Piceatannol 1				0,84459726
Piceatannol 2	[illegible]			0,85885735
Piceatannol 3	0,83167368			0,84808935
Piceatannol 4	0,84646096			0,72130864
(+)-Catechin 1				[illegible]
(+)-Catechin 2	0,88534546			0,86704589
(+)-Catechin 3	[illegible]			
(+)-Catechin 4				0,88052906
Mitomycin C 1				
Mitomycin C 2	0,85769626			0,55014771
Mitomycin C 3	0,64770134			0,59618138
Mitomycin C 4	0,83470456			0,69717153
Cytosine-ß-DArabinofuranosi de 1	[illegible]			
Cytosine-ß-DArabinofuranosi de 2	[illegible]			
Cytosine-ß-DArabinofuranosi de 3	[illegible]			
Cytosine-ß-DArabinofuranosi de 4				[illegible]
Daunorubicin Hydrochloride 1	4,57973132			[illegible]
Daunorubicin Hydrochloride 2	[illegible]			
Daunorubicin Hydrochloride 3	[illegible]			
Daunorubicin Hydrochloride 4				
Doxorubicin Hydrochloride 1				0,69883215
Doxorubicin Hydrochloride 2				1,24140002
Doxorubicin Hydrochloride 3	0,51782598			0,70579322
Doxorubicin Hydrochloride 4				0,83849377
Etoposide 1				
Etoposide 2				
Etoposide 3				[illegible]
Etoposide 4	0,82592957			[illegible]
Nocodazole 1	[illegible]			[illegible]
Nocodazole 2				[illegible]
Nocodazole 3				[illegible]
Nocodazole 4	[illegible]			
Quercetin Dihydrate 1	[illegible]			0,8087111
Quercetin Dihydrate 2	0,77346233			2,75693268
Quercetin Dihydrate 3	0,27316682			0,88205733
Quercetin Dihydrate 4				
Vinblastine Sulfate 1	[illegible]			0,76124932
Vinblastine Sulfate 2				0,88561233
Vinblastine Sulfate 3				
Vinblastine Sulfate 4	[illegible]			
